# Medical Waste Management: An Assessment of District-Level Public Health Facilities in Bangladesh

**DOI:** 10.7759/cureus.24830

**Published:** 2022-05-08

**Authors:** Hasnat Sujon, Taposh Kumar Biswas, Aklima Chowdhury, Mahbub Elahi Chowdhury

**Affiliations:** 1 Directorate General of Health Services, Ministry of Health and Family Welfare, Dhaka, BGD; 2 Health System and Population Studies Division, International Centre for Diarrhoeal Disease Research, Bangladesh, Dhaka, BGD

**Keywords:** mother and child welfare centre, healthcare waste, district hospital, medical waste, bangladesh

## Abstract

Background

Due to the huge patient load and different types of services, public health facilities produce a bulk of medical waste (MW) in Bangladesh. Improper disposal of MW increases the risk of infection among healthcare service personnel, patients, and attendants. To ensure quality services, this study aimed to assess the practices of MW management and quantify those to find out the shortcomings in the specific steps of waste management.

Methodology

As part of a larger interventional study, a facility assessment was conducted from February to April 2016 at a District Hospital (DH) and a Mother and Child Welfare Centre (MCWC) in one district. Non-participatory observation of MW management was done using a checklist that was developed following the Guideline for Medical Waste Management of Bangladesh. Scoring was applied for various activities of MW management performed in the study facilities.

Results

The overall scores for bin management, segregation, and collection of waste were 64.5%, 58.1%, and 62.0% in DH and 53.1%, 41.5%, and 48.0% in MCWC, respectively. The performance of operation theater in MCWC was the lowest among different corners (16.7% to 36.0%). Reusable waste was segregated poorly (32% in DH and 0% in MCWC), and almost none was shredded (4% in DH and 0% in MCWC). Waste was transported from in-house to out-house temporary storage area in an open bin without any trolley or specific route. The storage area was accessible to unauthorized persons, for example, a waste picker in DH. While DH segregated 84% of its infectious waste at the source, it eventually got mixed up with other waste in the storage area and delivered to the municipality to be dumped. MCWC could segregate only 40% of its infectious waste at the source and disposed of them using the pit method. Both the facilities disposed of sharp MW by open-air burning and liquid waste through sewerage without any treatment.

Conclusions

The performance of MW management was poor in both study facilities. Advocacy to the healthcare personnel and refresher training along with supportive supervision and monitoring may improve the situation. Moreover, a larger study is needed to find out the reasons behind such poor MW management.

## Introduction

Medical waste (MW) management is a growing concern worldwide, particularly in developing countries like Bangladesh [1]. As different health concerns compete for limited resources in developing countries, MW management remains a less prioritized business of healthcare facilities. Population growth, rapid urbanization, expansion of private healthcare facilities, and increasing use of disposable medical equipment are other factors contributing to this burden [2]. The total waste stream produced by healthcare delivery can be categorized as non-hazardous and hazardous waste. Although 75-90% of MW are non-hazardous, the remaining 10-25% contain pathogenic microorganisms and toxic chemicals, which require special treatment to mitigate the environmental and health risks [3]. Although the amount of MW that needs to be processed in any country is minute compared to the entire quantity of waste produced, if it remains untreated, the consequences are far too great. If this hazardous waste is dumped with other waste without proper treatment, the entire waste stream becomes a potential source of infection [4]. People in close proximity to this hazardous waste by generating, handling, or being exposed to it are vulnerable to serious diseases such as human immunodeficiency virus (HIV), hepatitis B, hepatitis C, etc. [3,5]. Globally, one out of three and in Bangladesh nine out of ten hospitals lack basic MW management services [6].

MW management is a systemic approach from the point of generation to its final disposal through effective segregation, handling, and treatment [7]. An effective MW management system is an integral part of infection prevention measures of a facility and is crucially associated with the quality of care and safety of both providers and patients along with the community [8]. The irony of the healthcare delivery system in developing countries like Bangladesh is that, while the healthcare facilities are entrusted to heal the sick and maintain wellness if proper interventions are not taken, the system itself can produce health hazards, thereby becoming a source of disease risk. Proper management of MW can minimize the risk of further burdens, both within and outside healthcare facilities, by limiting a definitive source of preventable infection [7]. If the healthcare delivery system is unable to address the urgent issue of MW management, channels of diseases and epidemic outbreaks would widen [9], thereby the healthcare situation would be “curing at the front door and poisoning at the back door” [10]. The importance of MW management is well-grounded and regarded as an integral part of the healthcare delivery process [11].

At the district level in Bangladesh, there is a District Hospital (DH) and a Mother and Child Welfare Centre (MCWC) under the Ministry of Health and Family Welfare. While all types of secondary healthcare services are provided by the DHs, the MCWCs provide only maternal and child health services along with family planning services. Thus, the structure, service availability, and human resources are quite distinct in these two types of facilities. However, both the facilities manage referred patients from sub-district and lower levels [12]. A Guideline for Medical Waste Management [11] has been developed by the Ministry of Health and Family Welfare for the healthcare facilities of Bangladesh; however, successful execution has remained a major concern.

Several studies have been conducted in Bangladesh to understand the MW management system. These studies focused on the quantification and final disposal method of MW and the knowledge of the staff on and barriers to efficient MW management [13-20]. To find an answer to the existing gap in MW management in Bangladesh, it is imperative to evaluate every step of MW treatment properly from its source to disposal. To the best of our knowledge in Bangladesh, until recently, no study thoroughly examined all steps of in- and out-house MW management practices and quantified the performance of healthcare facilities according to the standard guideline. The current study assessed the MW management practices in two district-level public healthcare facilities according to the Guideline for Medical Waste Management in Bangladesh. It also documented the existing gaps and shortcomings in the implementation of the MW management guideline. This article was previously posted to the Research Square preprint server on July 27, 2020 [21].

## Materials and methods

Study design

We conducted a cross-sectional facility assessment from February to April 2016 as part of a larger interventional study.

Study settings and population

The study was conducted in a 100-bed DH and a 20-bed MCWC located at a district headquarter in Bangladesh. Both study hospitals were secondary-level facilities. In both facilities, cleaners/ayas were assigned to perform waste management. We assessed waste management in the emergency room, labor and gynecology ward, operation theater (OT), pediatrics ward, pathology unit, and outdoor (antenatal and postnatal care room) of the study facilities separately. In the MCWC, separate emergency rooms, pediatrics wards, and pathology units were not available.

Data collection

To assess the waste management system, we developed a contextualized checklist (Appendices) based on the nature of MW likely to be generated in the study facilities following the Guideline for Medical Waste Management [11] of the Government of Bangladesh. Details of the implementation process of the waste management activities in different corners were collected through non-participatory direct observation using the checklist which included in-house and out-house facility waste management sections. Basic components of the in-house facility waste management section included bin management, segregation of waste, and waste collection from different wards/rooms. The basic components of out-house facility waste management were temporary waste storage area and final disposal of the waste. The temporary waste storage area was further divided into the transportation of waste to the storage area, management of the temporary waste storage area, and waste collection process by the municipality/authority.

A team consisting of two medical doctors, two trained interviewers, and one qualitative researcher was assigned to collect data from the designated hospitals. All members were trained thoroughly on the quality and ethical issues regarding data collection.

Scoring system

Individual scoring was done for different components of MW management and then an average score was calculated. For scoring bin management and waste collection from the wards/rooms, a full score was provided if the management of a particular room was maintained according to the guideline but failure to do so resulted in a deduction of the score. For example, if three bins among the recommended five were labeled, the maximum score was 5 and the score obtained was 3. For segregation of waste, a score was provided on a scale of 0 to 5 as only five types of waste were produced in both facilities. A score of 5 was given if the bins did not contain a mixture of waste. For partial segregation, provided score was lowered leading to 0 for all five types of waste being disposed of in the same-colored bin.

For components of out-house facility waste management, we observed the availability of equipment, logistics, and supplies along with practices.

Data analysis

The data were analyzed using SPSS version 21 (IBM Corp., Armonk, NY, USA). All entered data were then checked for inconsistency by applying logical conditions. If there was any inconsistency, necessary corrective measures were taken after comparing the entered data with those in the checklist. Percentage scoring was done separately for each component in every ward/room and an overall score was calculated for each component of waste management. For interpretation, we categorized >80% scores as high, 50-80% scores as moderate, and <50% scores as low. We accepted this arbitrary scoring based on a conventional grading system.

## Results

In-house facility waste management

**Figure 1:**
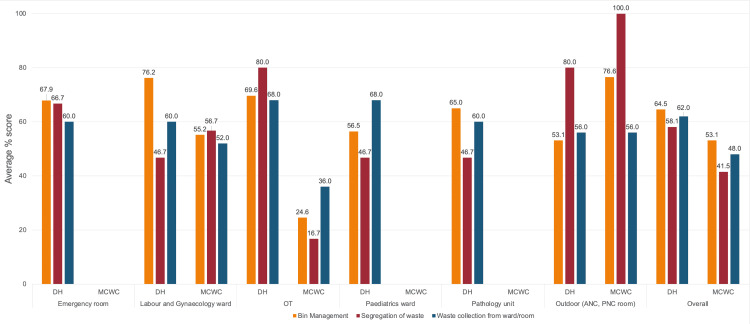
Average percentage score obtained in in-house facility waste management by different wards/rooms. DH = District Hospital; MCWC = Mother and Child Welfare Centre; OT = pperation theater; ANC = antenatal care; PNC = postnatal care

Overall scores in bin management were 64.5% in DH and 53.1% in MCWC. The highest score in bin management was obtained by the labor and gynecology ward (76.2%) in DH and outdoor (76.6%) in MCWC. Although the OT in MCWC obtained a score of 24.6%, other service areas of both DH and MCWC obtained moderate scores (53.1-76.6%) in bin management. In segregation of waste, the DH and MCWC obtained 58.1% and 41.5% scores, respectively. Although the outdoor MCWC obtained a score of 100%, the overall score was poor due to a very low score in the OT (16.7%). In the DH, the segregation of waste was better in OT and outdoor (both scored 80%) compared to the other service areas as each obtained a poor score (46.7% each). Scores in the collection of waste varied from 56% to 68% in DH and 36% to 56% in MCWC (Figure 1). A standard waste management guideline was available in both facilities.

Component-wise analysis revealed a wide variation among the scores of bin management in both facilities. In DH, though all bins were intact (neither broken nor leaked) and accessible to the patients, none were labeled and only 44% were closed with a lid. The scores in MCWC were more or less similar to the DH. The score on waste management-related “poster above the bin” was only 10% in DH and 0% in MCWC (Table 1).

**Table 1:** Average score (%) obtained in different segments of in-house facility waste management in DH and MCWC. DH = District Hospital; MCWC = Mother and Child Welfare Centre

Components of in-house facility waste management		Average score obtained			
		DH		MCWC	
		Score (n)	%	Score (n)	%
Bin management					
	Correct in placement	14 (16)	87.5	6 (9)	66.6
	Correct in color	25 (30)	83.3	11 (15)	73.3
	Closed with lid	11 (25)	44.0	9 (15)	60.0
	Neither leaked nor broken	30 (30)	100.0	11 (15)	73.3
	Cleanliness of bins	27 (30)	90.0	11 (15)	73.3
	Not exceeding capacity	29 (30)	96.6	11 (15)	73.3
	Color code written	20 (30)	66.6	8 (15)	53.3
	Color code maintained	18 (30)	60.0	9 (15)	60.0
	Labeled	0 (30)	0.0	0 (15)	0.0
	Accessible for patient	20 (20)	100.0	5 (5)	100.0
	Surrounding cleanliness	28 (30)	93.3	12 (15)	80.0
	Flip chart/poster available regarding infection prevention	8 (30)	26.6	2 (15)	13.3
	Waste management related poster above the bin	3 (30)	10.0	0 (15)	0.0
	Overall	233 (361)	64.5	95 (179)	53.1
Segregation of waste					
	General waste-keeping in black bin	25 (30)	83.3	10 (15)	66.6
	Sharp waste-keeping in red bin	23 (25)	92.0	5 (10)	50.0
	Infectious waste-keeping in yellow bin	21 (25)	84.0	4 (10)	40.0
	Reusable waste-keeping in green bin	8 (25)	32.0	0 (10)	0.0
	Liquid waste-keeping in a blue bin/bowl	12 (25)	48.0	8 (10)	80.0
	Reusable wastes shredding	1 (25)	4.0	0 (10)	0.0
	Overall	91 (155)	58.1	27 (65)	41.5
Waste collection from ward/room					
	Availability of an extra bin to replace the existing bin while emptying	0 (30)	0.0	0 (15)	0.0
	Proper emptying of bins	30 (30)	100.0	11 (15)	73.3
	Cleaning of bins after waste collection	27 (30)	90.0	10 (15)	66.6
	Cleaners wear protective materials	10 (30)	33.3	3 (15)	20.0
	Cleaners follow proper hand washing	26 (30)	86.6	12 (15)	80.0
	Overall	93 (150)	62.0	36 (75)	48.0

Segregation of waste was the most neglected component in both facilities. Among six components of segregation of waste, though the DH obtained more than 80% scores in general, sharp and infectious waste segregation, for the other three components, viz. segregation of reusable waste, and liquid waste and shredding of reusable waste, the scores were quite low ranging from 4% to 48%. The MCWC failed to obtain any score in segregation or shredding of reusable waste but scored 80% in segregation of liquid waste (Table 1).

Analysis of different components of waste collection from ward/room showed proper emptying of bins, cleaning after waste collection, and proper handwashing practice yielded moderate-to-high scores in both DH (100%, 90.0%, and 86.6%, respectively) and MCWC (73.3%, 66.6%, and 80.0%, respectively). Scores for personal protective equipment (PPE) wearing were very low in both facilities, and zero scores were noted in the availability of extra bins to be replaced with the existing bins (Table 1).

Out-house facility waste management

There was no trolley or handcart for carrying waste bins in any study facility for transportation of waste from in-house to the temporary storage area and the route of transportation was not separated. Among five bins, three in DH and one in MCWC were covered with a lid during transportation. Regarding management of the temporary waste storage area, among the advocated 11 components, DH could meet only four in contrast to eight components in MCWC. Although the location of the temporary storage area was far from the proximity of food preparation and had proper light with passive ventilation in both facilities, cleaning and firefighting equipment was unavailable in both facilities. The dedicated temporary waste storage area in DH had no good drainage system or water supply. Unauthorized persons, for example, scavengers and waste pickers, could easily get access to it. In contrast, though the temporary waste storage area of MCWC had a drainage system and water supply, they sometimes stored waste for more than 24 hours. However, unlike DH, unauthorized persons could not get access to it. Among the eight components of the waste collection process by municipality/authority, DH and MCWC followed two and three components, respectively. Although the DH showed better segregation in in-house waste management, all of these segregated waste got mixed in the temporary storage area and the municipality/authority collected them without any color code, whereas MCWC supplied the segregated waste (though all of the segregation was not done properly) to municipality/authority as per the color code. In none of the facilities, cleaners used any protective materials (e.g., hand gloves, shoes, long sleeve shirts). Though drivers’ areas were separated from the waste carrying area, none of the vans were covered or under lock or properly cleaned every day after carrying waste (Table 2).

**Table 2:** Out-house facility waste management in the temporary waste storage area in DH and MCWC. ^a^Three of five bins were covered with a lid; ^b^one of five bins was covered with a lid. DH = District Hospital; MCWC = Mother and Child Welfare Centre

Components of out-house facility waste management of temporary waste storage area		DH	MCWC
Transportation of waste to temporary waste storage area			
	Trolley available for carrying waste bin	No	No
	Waste bin covered with a lid during transportation	Partially^a^	Barely^b^
	Specific route for transportation of waste	No	No
Temporary waste storage area management			
	Impermeable, hard-standing floor with a good drainage system	No	Yes
	Water supply for cleaning purpose	No	Yes
	Equipped with cleaning equipment, sand, and firefighting equipment	No	No
	Inaccessibility to unauthorized persons, animals, and insects	No	Yes
	Proper light, passive ventilation	Yes	Yes
	Location of the area far from the proximity of food preparation area	Yes	Yes
	Bins are covered with a lid	No	Yes
	Under lock and key/door is closed	No	Yes
	Easy access for van/truck to take waste	Yes	Yes
	Waste not stored for more than 24 hours	Yes	No
	For emergency waste stored for more than 24 hours, informing higher authority and ensuring no harm to others	-	No
Waste collection process by municipality/authority			
	Collection of waste as per the color code from facility	No	Yes
	Collection of waste in a covered van	No	No
	The driver area totally separated from the waste-carrying area	Yes	Yes
	Transport within a short time	Yes	Yes
	Cleaners wear protective cloths	No	No
	Waste in the van in under lock	No	No
	Van is air conditioned to keep waste for long time	No	No
	Van is properly cleaned every day after carrying waste	No	No

Final disposal of medical waste

The Guideline for Medical Waste Management of Bangladesh prescribed different disposal methods for different segregated waste. In both DH and MCWC, the final disposal of MW was more or less similar. An incinerator was not available in any of the facilities. Both facilities followed open-air burning for sharp wastes. MCWC disposed of infectious wastes by pit method but as they got mixed with other waste in DH, they were dumped as general waste. Both facilities used public dustbins of the municipality to dispose of general waste and sewerage systems to dispose of liquid waste without any treatment. There was no system for recycling reusable waste (Table 3).

**Table 3:** Final methods of medical waste disposal in DH and MCWC. DH = District Hospital; MCWC = Mother and Child Welfare Centre

Type of waste	Recommended method(s) for waste disposal	Scenario in	
		DH	MCWC
Sharp waste	Incinerator	X	X
	Open-air burning (making a hole)	√	√
	Pit method	√	X
Infectious waste	Pit method	X	√
	Deep burial	X	X
	Incinerator	X	X
Recyclable waste	Shredded before disposal	X	X
	Reuse by disinfection	X	X
	Reuse by autoclave	X	X
General waste	Dumping in municipality public dustbin	√	√
Liquid waste	Sewerage system	√	√

## Discussion

The practices of MW management in both the study facilities were clearly insufficient and poor. While every recommended procedure of MW management needs to be followed step by step, on average, both facilities performed only about half of the recommended activities. The inadequacy in performing MW treatment was quite distinct between the facilities. While MCWC grossly lacked in-house facility waste management, DH performed relatively poorly in out-house facility waste management. The absence of labeling, written color codes, and instructions may have augmented the improper use of colored bins. Inadequate segregation of waste at the source and absence of shredding of reusable items, along with defective waste transportation and management of temporary waste storage areas, made the whole waste management system injurious to human and environmental health. The absence of PPE led to a higher risk to the personnel involved in the MW management system.

The initial stage of waste management is bin management which was moderate in both facilities. Six color-coded bins are recommended for healthcare facilities in Bangladesh: black for general waste, red for sharp objects, yellow for infectious waste, green for reusable waste, blue for liquid waste, and silver for radioactive waste [11]. As no radioactive waste was generated in any of the study facilities, a silver bin was not there. In developing countries like Bangladesh, usually marginalized, uneducated, and economically vulnerable personnel are engaged in waste management [2]. The absence of labeling and inadequate instruction (flip chart/poster) coupled with the absence of training may contribute to the mismanagement of the bins by the cleaners which has been reported by other studies [15,16,22]. In addition, people attending the public healthcare facilities of Bangladesh are largely illiterate. Even educated patients and their attendants may not know the significance of color code without instruction. Accessible bins without written color codes and instructions may also encourage people to indiscriminately dispose of waste which can make the segregation of waste at the source a challenging task [23]. While transporting the bins to the temporary waste storage area to be emptied, the vacant positions are supposed to be replaced by extra bins which both facilities lacked. As a result, people had to dispose of waste openly or in left-out bins without any regard to color code.

An MW management system with inadequate segregation of infectious waste can be translated as a defective system on the whole. Waste segregation at the source through proper use of bins is essential to avoid mixing infectious waste with other non-hazardous waste, which acts as a strong barrier against making the entire waste stream hazardous [3]. We observed poor infectious waste segregation, particularly in MCWCs. As the entire infectious waste could not be segregated, other non-hazardous waste was mixed with the unsegregated infectious waste. Segregation and shredding of reusable waste was another neglected issue in both facilities. An effective waste management system is supposed to follow the 3R of Reduce, Recycle, and Reuse [2]. Whereas recycling and reusing some waste like paper and hardboard are economically beneficial, shredding of certain reusable waste, for example, saline set and syringes provides protection against the collection and reselling of equipment.

As OTs produce one-third of all MW generated in a facility [24], the shortcomings in all components of in-house facility waste management are of particular importance. Being the center of life-saving surgeries, ineffective bin management, waste segregation, and collection in OTs may cause intraoperative and postoperative infection in patients.

A separate route is recommended to be used at a specific time of the day to carry covered waste to the temporary waste storage area in a designated trolley or handcart to minimize the risk of exposure to hospital staff, patients, and visitors, none of which was maintained in both facilities. Waste transportation in open bins may result in spillage. It is recommended that cleaners should use masks, service gloves, long sleeve shirts, plastic aprons, and boot shoes while conducting their service. The cleaners, however, handled and disposed of the waste without any PPE which exposed them to a high risk of infections [25]. Unrestricted access of unauthorized persons, for example, waste pickers and scavengers is another major issue. As facilities do not shred reusable items, for example, syringes and saline bags, waste pickers can collect them from the temporary waste storage area. They handle these items with bare hands and sell them in the local market [17]. If improperly treated and reused, this equipment may cause life-threatening diseases including hepatitis B, hepatitis C, and HIV/AIDS. Persons receive 2.88 injections per year in developing and transitional countries like Bangladesh, of which 5.5% are administered with a reused syringe [26]. Several years ago, a black market for these infected items was discovered in Gujarat in India which eventually claimed more than 50 lives [27].

We observed the global concern of “waste is sometimes treated without being segregated, and segregated waste is often not treated” [6] in both facilities, although the problem was quite distinct between the facilities. The DH segregated most of its waste at the point of generation, but the cleaners delivered all the waste mixed together to the municipality with no regard to color code except the sharps. This signifies a lack of knowledge, rather than a lack of will, among the cleaners. MCWC, however, could segregate only a limited amount of its waste at the source and delivered it to the municipality as per the color code. While their effort should be commended, as only less than half of the infectious waste was segregated at the source, other waste was also mixed with infectious waste. The municipality/authority that collected these wastes for their disposal also did not follow most of the guidelines. As a result, the whole lot of waste became potentially infectious.

While both the facilities had procedures in place to dispose of sharp and general waste, they paid little attention to the proper disposal of infectious or recyclable waste. Both facilities usually used the municipality dustbin to dump the general waste. Infectious waste was dumped in the municipality dustbin along with the general waste due to the absence of procedures to dispose of infectious waste, low waste segregation at the source, and eventual mixing of wastes in the temporary waste storage area. This type of erratic handling and indiscriminate disposal of MW increase the risk of human contact with hazardous waste and expose the entire community to a risk of environmental tragedy. While incinerator remains a viable option for waste disposal (although criticized for its harmful effects on the environment) in developing countries including Bangladesh, none of these facilities were equipped with the facility. The dumping of infectious and liquid waste without any treatment has long been discussed for their detrimental effects on the environment [2,5,13,22].

The need for proper waste management to protect providers’ and clients’ health and prevent environmental tragedies is overwhelming. Numerous studies conducted in Asia and Africa have looked into the issue of the underlying causes of improper waste management [2,9,15]. The economic condition of a country despite policy has been directly linked with the level of management of MW [1]. Lack of training is another major barrier for effective waste management. Incorporating all staff of a facility including doctors, nurses, paramedics, and cleaners was suggested for a whole-of-the-facility approach to waste management. Facilities should emphasize waste reduction as this equals to less workload [2]. Lack of human resources in the healthcare facilities along with managerial weakness, suboptimal supervision, and confusion over duty and responsibility allocation are other causative factors for inefficient management [23]. While the need for managerial strength and training should be in the central column to implement proper MW management, noncompliance, and neglect of some facilities regarding this issue have also been reported [28]. Initiatives should be taken immediately to improve the motivation for the staff, monitoring, and supervision system to implement waste management guidelines at every stage. Although MW management is not completely risk free, some low-cost investments can bring good results, for example, awareness building among people attending hospital, supply of PPE and equipment, use of instructional posters, etc. [1]. All cleaners should be screened and vaccinated. The final waste disposal method should be developed considering the safety, cost, and local technology [4,13].

Study limitations

The study was conducted only in two secondary-level public healthcare facilities in Bangladesh, and the background reasons for improper management were not explored. As all healthcare facilities of a single tier do not follow a standard waste management system in Bangladesh despite the presence of a national guideline, it is difficult to generalize the scenario even in a single tier. Furthermore, this study was conducted several years ago; therefore, the current scenario may not be exactly the same. Nontheless, this study tried to identify the areas to improve in everyday practice and provide a snapshot of the waste management scenario in a developing country. Further qualitative studies are needed for exploring the reasons behind the current situation and to find out interventions to address the gaps.

## Conclusions

MW involves a potential source of injury, infection, and environmental damage. Although the consequences of improper management of MW are highly negative, only a small percentage of MW needs special consideration. The study findings do not support the proper following of the Guideline for Medical Waste Management in public facilities in Bangladesh. Most of the gaps identified can be addressed by proper training and motivation of the hospital staff, purchase of some inexpensive equipment, and awareness. Making administrative and institutional mechanisms in place with effective monitoring and supervision may alter the situation to safeguard environmental and human health. In addition, necessary budget allocation for continuous advocacy and periodic refresher training of the relevant staff should be ensured by the authority for better MW management in health facilities.

## Appendices

**Table 4:** Checklist for in-house facility waste management.

Area	Topic	Indicators/Variables/Activities						Completed = 3, Mostly completed = 2, partially completed = 1, No = 0, N/A = 9	Comment	
1. Emergency room	A. Bin management (placement of bins):	1. Placed bins are correct in color according to the type of service delivery (give a tick mark)								
			Black	Red	Yellow	Green	Blue			
		Should be present		√	√	√	√			
		Available								
		2. Bins are closed with a lid								
		Black	Red	Yellow	Green	Blue				
		3. Placed bins are not leaking or broken										
		Black	Red	Yellow	Green	Blue					
		4. Placed bins are clean										
		Black	Red	Yellow	Green	Blue				
		5. Wastes of the container are not exceeding three-quarters of its capacity										
		Black	Red	Yellow	Green	Blue					
		6. Written on the bin to dispose of type of waste following the color code										
		Black	Red	Yellow	Green	Blue				
		7. Color code is maintained to dispose of wastes type										
		Black	Red	Yellow	Green	Blue					
		8. Labeling of the bin with number or ward name										
		Black	Red	Yellow	Green	Blue				
		9. Bins are accessible for patients										
		10. Surrounding cleanliness of the bins is maintained								
		11. Any flip chart or poster regarding infection prevention or waste management available								
		12. Waste management-related poster is available above the bins								
		B: Segregation of waste according to classification	13. General wastes are kept in the black bin								
			14. Sharp wastes are kept in the red bin								
			15. Infectious wastes are kept in the yellow bin								
			16. Reusable wastes are kept in the green bin								
			17. Liquid wastes only kept in the blue bin/bowl								
			18. Reusable wastes are shredded/pieces in the bin								
		C. Waste collection from ward/room	19. Use an extra bin to be replaced with an existing bin								
	20. Emptying of bins is done properly								
	Black		Red	Yellow	Green	Blue				
	21. Bins are cleaned after waste collection										
	Black		Red	Yellow	Green	Blue				
	22. Waste collectors wear protective materials										
	Should wear		Mask	Service gloves	Long-sleeved shirt	Plastic apron			Boot shoe	
	Seen to wear									
	23. Proper handwashing is practiced by service providers after waste handling								
	D. Training in waste management		24. Number of trained persons in medical waste management								
			Designation	Total staff	Trained among them	Trained within 1 year = 1, more than 1 year= 2					
		Medical officers/Consultant								
		Staff nurse								
		Aya/ward boy/MLSS								
		Cleaner								
		25. Sessions are organized by the staff nurses indoors on the roles and responsibilities of patients and attendants in waste management								
	2. Labor room and gynecology ward	A. Bin management (placement of bins):	1. Placed bins are correct in color according to the type of service delivery (give tick mark)								
				Black	Red	Yellow	Green	Blue			
			Should be present	√	√	√	√	√			
			Available								
			2. Bins are closed with a lid								
			Black	Red	Yellow	Green	Blue				
			3. Placed bins are not leaking or broken										
			Black	Red	Yellow	Green	Blue					
			4. Placed bins are clean										
Black			Red	Yellow	Green	Blue				
5. Wastes of the container are not exceeding three-quarters of its capacity										
Black			Red	Yellow	Green			Blue		
6. Written on the bin to dispose of type of waste following the color code										
Black			Red	Yellow	Green	Blue				
7. Color code is maintained to dispose of wastes type										
Black			Red	Yellow	Green			Blue		
8. Labeling of the bin with number or ward name										
Black			Red	Yellow	Green	Blue				
9. Bins are accessible for patient										
10. Surrounding cleanliness of the bins is maintained								
11. Any flip chart or poster regarding Infection prevention or waste management available								
12. Waste management related poster is available above the bins								
B: Segregation of waste according to classification			13. General wastes are kept in the black bin								
			14. Sharp wastes are kept in the red bin								
			15. Infectious wastes are kept in the yellow bin								
			16. Reusable wastes are kept in the green bin								
			17. Liquid wastes only kept in the blue bin/bowl								
			18. Reusable wastes are shredded/pieces in the bin								
C. Waste collection from ward/room			19. Use an extra bin to be replaced with an existing bin								
		20. Emptying of bins is done properly								
		Black	Red	Yellow	Green	Blue				
		21. Bins are cleaned after waste collection										
		Black	Red	Yellow	Green	Blue					
		22. Waste collectors wear protective materials										
		Should wear	mask	Service gloves	Long-sleeved shirt	Plastic apron	Boot shoe			
		Seen to wear								
		23. Proper handwashing is practiced by service providers after waste handling								
		D. Training in waste management	24. Number of trained persons in medical waste management								
			Designation	Total Staff	Trained among them	Trained within 1 year = 1, more than 1 year = 2					
Medical officers/Consultant										
Staff nurse										
Aya/ward boy/MLSS										
Cleaner										
25. Sessions are organized by the staff nurses indoors on the roles and responsibilities of patients and attendants in waste management								
3. Operation theater room		A. Bin management (placement of bins):	1. Placed bins are correct in color according to the type of service delivery (give tick mark)								
				Black	Red	Yellow	Green	Blue			
			Should be present		√	√	√	√			
			Available								
			2. Bins are closed with a lid								
			Black	Red	Yellow	Green	Blue				
			3. Placed bins are not leaking or broken										
			Black	Red	Yellow	Green	Blue					
			4. Placed bins are clean										
	Black		Red	Yellow	Green	Blue				
	5. Wastes of the container are not exceeding three-quarters of its capacity										
	Black		Red	Yellow	Green	Blue				
	6. Written on the bin to dispose of the type of waste following the color code										
	Black		Red	Yellow	Green	Blue				
	7. Color code is maintained to dispose of wastes type										
	Black		Red	Yellow	Green	Blue				
	8. Labeling of the bin with number or ward name										
	Black		Red	Yellow	Green	Blue				
	9. Bins are accessible for patient										
	10. Surrounding cleanliness of the bins is maintained								
	11. Any flip chart or poster regarding infection prevention or waste management available								
	12. Waste management related poster is available above the bins								
	B: Segregation of waste according to classification		13. General wastes are kept in the black bin								
			14. Sharp wastes are kept in the red bin								
			15. Infectious wastes are kept in the yellow bin								
			16. Reusable wastes are kept in the green bin								
			17. Liquid wastes only kept in the blue bin/bowl								
			18. Reusable wastes are shredded/pieces in the bin								
	C. Waste collection from ward/room		19. Use an extra bin to be replaced with an existing bin								
		20. Emptying of bins is done properly								
		Black	Red	Yellow	Green	Blue				
		21. Bins are cleaned after waste collection										
		Black	Red	Yellow	Green	Blue					
		22. Waste collectors wear protective materials										
		Should wear	mask	Service gloves	Long-sleeved shirt	Plastic apron	Boot shoe			
		Seen to wear								
		23. Proper handwashing is practiced by service providers after waste handling								
		D. Training in waste management	24. Number of trained persons in medical waste management								
			Designation	Total Staff	Trained among them	Trained within 1 year = 1, more than 1 year = 2					
	Medical officers/Consultant									
	Staff nurse									
	Aya/ward boy/MLSS									
	Cleaner									
	25. Sessions are organized by the staff nurses indoors on the roles and responsibilities of patients and attendants in waste management								
	4. Pediatrics ward	A. Bin management (placement of bins):	1. Placed bins are correct in color according to the type of service delivery (give tick mark)								
				Black	Red	Yellow	Green	Blue			
			Should be present	√	√	√	√	√			
			Available								
			2. Bins are closed with a lid								
			Black	Red	Yellow	Green	Blue				
			3. Placed bins are not leaking or broken										
			Black	Red	Yellow	Green	Blue					
			4. Placed bins are clean										
Black			Red	Yellow	Green	Blue				
5. Wastes of the container are not exceeding three-quarters of its capacity										
Black			Red	Yellow	Green			Blue		
6. Written on the bin to dispose of the type of waste following the color code										
Black			Red	Yellow	Green	Blue				
7. Color code is maintained to dispose of wastes type										
Black			Red	Yellow	Green			Blue		
8. Labeling of the bin with number or ward name										
Black			Red	Yellow	Green	Blue				
9. Bins are accessible for patient										
10. Surrounding cleanliness of the bins is maintained								
11. Any flip chart or poster regarding infection prevention or waste management available								
12. Waste management related poster is available above the bins								
B: Segregation of waste according to classification			13. General wastes are kept in the black bin								
			14. Sharp wastes are kept in the red bin								
			15. Infectious wastes are kept in the yellow bin								
			16. Reusable wastes are kept in the green bin								
			17. Liquid wastes only kept in the blue bin/bowl								
			18. Reusable wastes are shredded/pieces in the bin								
C. Waste collection from ward/room			19. Use an extra bin to be replaced with an existing bin								
		20. Emptying of bins is done properly								
		Black	Red	Yellow	Green	Blue				
		21. Bins are cleaned after waste collection										
		Black	Red	Yellow	Green	Blue					
		22. Waste collectors wear protective materials										
		Should wear	mask	Service gloves	Long-sleeved shirt	Plastic apron	Boot shoe			
		Seen to wear								
		23. Proper handwashing is practiced by service providers after waste handling								
		D. Training in waste management	24. Number of trained persons in medical waste management								
			Designation	Total staff	Trained among them	Trained within 1 year = 1, more than 1 year = 2					
Medical officers/Consultant										
Staff nurse										
Aya/ward boy/MLSS										
Cleaner										
25. Sessions are organized by the staff nurses indoors on the roles and responsibilities of patients and attendants in waste management								
								5. Pathology	A. Bin management (placement of bins):	1. Placed bins are correct in color according to the type of service delivery (give tick mark)									
		Black	Red	Yellow	Green	Blue											
Should be present			√	√	√	√											
Available																	
2. Bins are closed with a lid											
Black		Red	Yellow	Green	Blue							
3. Placed bins are not leaking or broken													
Black		Red	Yellow	Green	Blue							
4. Placed bins are clean													
Black		Red	Yellow	Green	Blue							
5. Wastes of the container are not exceeding three-quarters of its capacity													
Black	Red	Yellow	Green	Blue							
6. Written on the bin to dispose of the type of waste following the color code													
Black	Red	Yellow	Green	Blue							
7. Color codes are maintained to dispose of wastes type													
Black	Red	Yellow	Green	Blue							
8. Labeling of bins with number or ward name													
Black	Red	Yellow	Green	Blue							
9. Bins are accessible for patients													
10. Surrounding cleanliness of the bins is maintained											
11. Any flip chart or poster regarding infection prevention or waste management available											
12. Waste management related poster is available above the bins											
B: Segregation of waste according to classification	13. General wastes are kept in the black bin											
	14. Sharp wastes are kept in the red bin											
	15. Infectious wastes are kept in the yellow bin											
	16. Reusable wastes are kept in the green bin											
	17. Liquid wastes only kept in the blue bin/bowl											
	18. Reusable wastes are shredded/pieces in the bin											
C. Waste collection from ward/room	19. Use an extra bin to be replaced with an existing bin											
	20. Emptying of bins is done properly										
	Black	Red	Yellow	Green	Blue						
	21. Bins are cleaned after waste collection												
	Black	Red	Yellow	Green	Blue						
	22. Waste collectors wear protective materials												
	Should wear	mask	Service gloves	Long-sleeved shirt	Plastic apron	Boot shoe					
	Seen to wear										
	23. Proper handwashing is practiced by service providers after waste handling										
	D. Training in waste management	24. Number of trained persons in medical waste management										
		Designation	Total staff	Trained among them	Trained within 1 year = 1, more than 1 year = 2							
Medical officers/Consultant											
Staff nurse											
Aya/ward boy/MLSS											
Cleaner											
25. Sessions are organized by the staff nurses indoors on the roles and responsibilities of patients and attendants in waste management										
6. Outdoor (ANC, PNC room)	A. Bin Management (placement of bins):	1. Bins available at										
		status	Entrance of hospital’s gate	Patient waiting area	Landing area of the staircase	In the long corridor at 50-meter distance						
		Should be present	√	√	√	√						
		Available										
		2. Placed bins are correct in color according to the type of service delivery (give tick mark)										
			Black	Red	Yellow	Green	Blue					
		Should be present	√									
		Available										
		3. Bins are closed with a lid										
		4. Placed bins are not leaking or broken									
		5. Placed bins are clean									
		6. Wastes of the container are not exceeding three-quarters of its capacity									
		7. Written on the bin to dispose of type of waste following color code									
		8. Color codes are maintained to dispose of wastes type									
		9. Labeling of the bin with number or ward name									
		10. Bins are accessible for patient									
		11. Surrounding cleanliness of the bins is maintained									
		12. Any flip chart or poster regarding infection prevention or waste management available									
		13. Waste management related poster is available above the bins									
	B. Segregation and waste collection	14. General wastes are kept in a black bin									
		15. Use an extra bin to be replaced with an existing bin									
		16. Emptying of bins is done properly									
		17. Bins are cleaned after waste collection									
		Should wear	mask	Service gloves	Long-sleeved shirt	Plastic apron	Boot shoe				
		seen to wear									
		18. Waste collectors wear protective materials									
		19. Proper handwashing is practiced by service providers after waste handling									
		20. Sessions are organized by the staff nurses indoors on the roles and responsibilities of patients and attendants in waste management									

**Table 5:** Checklist for out-house facility waste management and final disposal of the waste.

Area	Topic	Indicators/Variables/Activities	Yes = 1, No = 0, N/A = 9	Comment
Temporary waste storage area	Transportation of waste to the temporary waste storage area	1. Trolleys are available for carrying waste bin		
		2. Waste bins are covered with a lid during transportation		
		3. Selected route is used for waste carrying		
	Temporary waste storage room management	4. Impermeable, hard-standing floor with a good drainage system		
		5. Water supply for cleaning purposes		
		6. Equipped with cleaning equipment, sand, and fire fitting equipment and reagents		
		7. Inaccessibility for unauthorized persons, animals, and insects		
		8. Proper light, passive ventilation		
		9. Situation of the room within the proximity to the food preparation area		
		10. Covered with a lid		
		11. Under lock and key/door is closed		
		12. Easy access for van/truck to take wastes		
		13. Waste not stored more than 24 hours		
		14. For emergency waste stored for more than 24 hours, it is informed to higher authorities and ensured that it is harmful to others		
Ways of Medical Waste disposal	Incineration	15. Waste disposed of by incinerator? Yes = 1, No = 0		
		15.a. Types of incinerators		
		a. Drum incinerator = 1		
		b. Brick-made incinerator = 2		
		c. Single chambered = 3		
		d. Double chambered = 4		
		c. N/A = 9		
	Open-air burning	16. Waste disposed of by open-air burning? Yes = 1, No = 0		
		16.a Types of open-air burning		
		a. Make a hole = 1		
		b. Burning (direct) safety box (EPI) = 2		
		c. Surrounding brick wall = 3		
		c. N/A = 9		
	Sharp wastes disposes by Pit method	17. Sharp waste disposed of by the pit method? Yes = 1, No = 0		
		17.a. Types of pit		
		a. Deep hole pit =1		
		b. Concrete pit = 2		
		c. N/A = 9		
		17. b. Pit is covered with a lid?		
		Yes = 1, No = 0		
	Infectious waste disposes by Pit method	18. Infectious waste disposed of by the pit method? Yes = 1, No = 0		
		18. a. Types of pit		
		a. Deep hole pit = 1		
		b. Concreted pit = 2		
		c. N/A = 9		
		18.b. Pit is covered with a lid: Yes = 1, No = 0		
	Deep burial method	19. Waste disposed of by deep burial: Yes = 1, No = 0		
Out-house medical waste management	Pourosova/City corporation	20. Collection of waste (as per the color code) from the facility		
		21. Collection of waste in a covered van		
		22. The driver area totally separated from the waste carrying area		
		23. Transport within a short time		
		24. Cleaners wear protective cloths		
		25. Waste in the van is under lock		
		26. Van is air-conditioned to keep waste for a long time		
		27. Van is cleaned properly every day after carrying waste		
Waste management committee	Activity	28. Waste Management Committee available in the facility		
		29. Last meeting conducted by the committee?		
		Mention the date: _____________		
		Main issues are discussed:		
		a. _____________________________________		
		b. _____________________________________		
		c. _____________________________________		
